# Atrial conduction explains the occurrence of the P‐wave dispersion phenomenon, but weakly

**DOI:** 10.1002/joa3.12444

**Published:** 2020-10-15

**Authors:** Raimundo Carmona Puerta, Elibet Chávez González, Magda Alina Rabassa López‐Calleja, Elizabeth Lorenzo Martínez, Juan Miguel Cruz Elizundia, Gustavo Padrón Peña, Fernando Rodríguez González

**Affiliations:** ^1^ Department of Electrophysiology and Arrhythmology Cardiovascular Hospital "Ernesto Guevara" Santa Clara City Cuba; ^2^ Chief Professor in Cardiology Cardiovascular Hospital "Ernesto Guevara" Santa Clara City Cuba; ^3^ Department of Physiology Medical University of Villa Clara Santa Clara City Cuba

**Keywords:** atrial conduction time, electrophysiological study, maximum P‐wave duration, P‐wave dispersion

## Abstract

**Background:**

P‐wave dispersion (PWD) is believed to be caused by inhomogeneous atrial conduction. This statement, however, is based on limited little solid evidence. The aim of this study was to determine the relationship between atrial conduction and PWD by means of invasive electrophysiological studies.

**Methods:**

Cross‐sectional study in 153 patients with accessory pathways and atrioventricular node reentry tachycardia (AVNRT) undergoing an electrophysiological study. Different atrial conduction times were measured and correlated with PWD.

**Results:**

Only the interatrial (P‐DCS) and left intra‐atrial conduction times (ΔDCS‐PCS) showed a significant correlation with PWD, but this correlation was weak. Multivariate linear regression analysis determined that both P‐DCS (β = 0.242; *P* = .008) and ΔDCS‐PCS (β = 0.295; *P* < .001) are independent predictors of PWD. Performing the multivariate analysis for arrhythmic substrates, it is observed that only ΔDCS‐PCS continued to be an independent predictor of PWD. Analysis of the receiver operating characteristic curves showed that regardless of the types of arrhythmic substrates, PWD discriminates significantly, but moderately, to patients with P‐DCS and ΔDCS‐PCS ≥75 percentile.

**Conclusions:**

Interatrial and intraleft atrial conduction times were directly and significantly correlated with PWD, but only weakly, and were independent predictors of PWD. In general, PWD correctly discriminates patients with high values in interatrial and intraleft atrial conduction times, but moderately. This is maintained in cases with accessory pathways; however, in patients with AVNRT it only does so for intraleft atrial conduction times. Interatrial and intraleft atrial conduction times weakly explains PWD.

## Graphical abstract

P‐wave dispersion (PWD) is considered by many to be an electrocardiographic parameter originating from regional differences in atrial conduction. The aim of this study was to determine the relationship between atrial conduction and PWD by means of invasive electrophysiological studies. Interatrial and intra left atrial conduction times were directly and significantly correlated with PWD, but weakly, and were independent predictors of PWD.

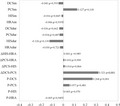

## 1 INTRODUCTION

In 1998 Dilaveris et al^1^ proposed the use of P‐wave dispersion (PWD) as a predictor of atrial fibrillation. Two main theories have been proposed to explain the origin of PWD. The local theory and the global theory.^2^ The local theory is the most widely disseminated theory. It argues that atrial zones with different conduction velocities give rise to P waves of different durations throughout the 12 leads of the electrocardiogram.^3^ Most connoisseurs of this topic accept that PWD reflects prolonged, inhomogeneous, and anisotropic distribution of connections between myocardial fibers resulting in discontinuous anisotropic propagation of sinus impulses, as well as, inhomogeneous and discontinuous atrial conduction.^4^ However, there are no studies specially designed to test this theory and those that approach the subject study atrial conduction through noninvasive methods. Therefore, we set out to determine the relationship between atrial conduction and PWD through invasive electrophysiological studies.

## 2 METHODS

A cross‐sectional study was carried out in 153 patients (mean age 39.53 ± 14.36; range 18‐70 years). The cases were randomly selected from a study population of 286 patients with a clinical history of palpitations and a confirmed diagnosis of atrioventricular node reentry tachycardia (AVNRT) or accessory pathways, who underwent electrophysiological study and endocardial ablation at the service of Cardiac Electrophysiology of the Cardiovascular Hospital "Ernesto Guevara" from Santa Clara city, Cuba, between June 2017 and February 2020. The electrophysiological study was performed after at least 6‐8 hours fasting and without antiarrhythmic medication, at least for 5 or more half‐lives of the drug. All subjects were assessed by transthoracic echocardiography for exclusion of cardiac anomaly.

Exclusion criteria were: (a) Had >2 electrocardiographic leads that did not allow measurement of the P wave and/or any channel of the atrial intracavity records with poor signal quality. (b) Permanent ventricular preexcitation (excluded because of the difficulty of measuring the P‐wave offset).

### 2.1 Study variables

The following general variables were taken into account: age, sex, comorbidities, body weight, and occurrence of atrial fibrillation during the electrophysiological study. Atrial fibrillation was considered to be present if paroxysms lasting >10 seconds were documented.

The electrocardiographic parameters studied included:

Heart rate: measured from R‐R interval, at time of the P wave and electrophysiological measurements.

Maximum P‐wave duration (PMax): P wave of greater duration in any of the 12 leads of the electrocardiogram.

Minimum P‐wave duration: P wave of shortest duration in any of the 12 leads of the electrocardiogram.

**FIGURE 1:**
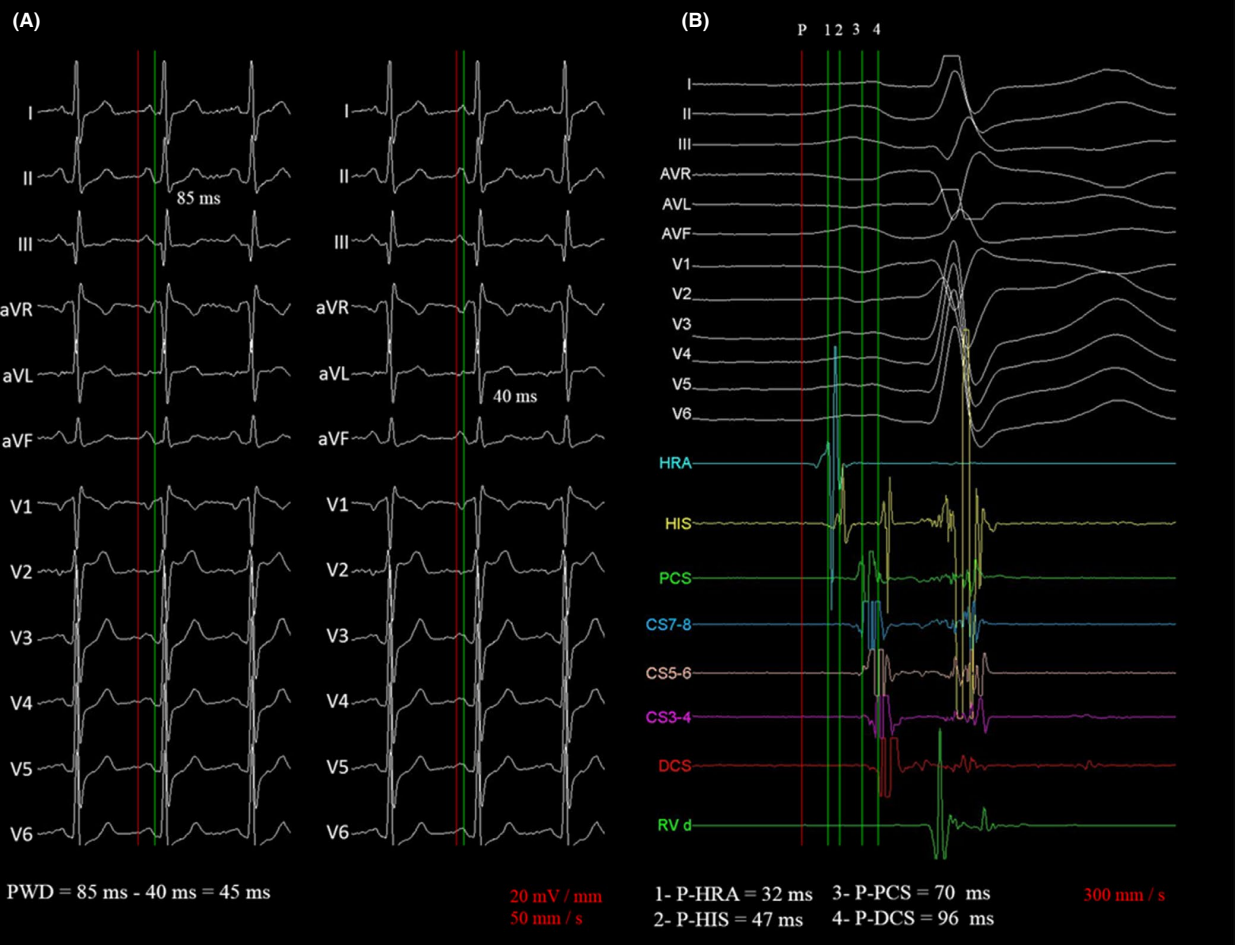
Example of measurement methods in a 68‐year‐old male patient diagnosed with atrioventricular nodal reentrant tachycardia and previous history of systemic hypertension. (A) Method used for the measurement of P‐wave dispersion using a 12‐lead simultaneous recording and hand‐held caliper. Pmax and Pmin were measured in the same beat to avoid the phenomenon of P‐wave lability over time. (B) Method used for the measurement of atrial conduction times (P‐A intervals). DCS, distal coronary sinus; HIS, atrial electrogram in the His bundle recording; HRA, high right atrium; P, earliest onset of the P wave; PCS, proximal coronary sinus; Pmax, maximum P‐wave duration; Pmin, minimum P‐wave duration; PWD, P‐wave dispersion

P‐wave dispersion (PWD): determined by subtracting the minimum P‐wave duration from the maximum P‐wave duration in any of the 12 standard leads of the electrocardiogram (Figure 1A).

Up to three consecutive P waves were measured in each of 12 leads and averaged. Electrocardiographic records were obtained at a calibration of 20 mm/mV and a sweep speed of 50 mm/s. The onset of the P wave is defined as the point of first detectable upward or downward slope from the isoelectric line for positive or negative waveforms, respectively. Return to the isoelectric line is considered as the end of the P wave. If the start or end of the P wave was not clearly defined, that lead was excluded. All electrocardiographic measurements were expressed in milliseconds.

The electrophysiological parameters studied included:

P‐high right atrium (HRA) interval: Measured from the onset of the P wave to the onset of the earliest reproducible rapid deflection of the atrial electrogram in the high right atrium, recorded with the distal pair of a BIOTRONIK quadripolar catheter (interelectrode distance of the same pair, 5 mm; distance between pairs, 10 mm). This parameter was accepted as a measure of the intraright atrial conduction time from the sinus node to the respective recording area (Figure 1B).

P‐HIS interval: Measured from the onset of the P wave to the onset of the earliest reproducible rapid deflection of the atrial electrogram in the His bundle recording. The local bipolar electrogram was obtained with the distal poles of a quadripolar BIOTRONIK catheter (inter electrode distance of the same pair, 5 mm; distance between pairs, 10 mm). This parameter was accepted as a measure of the intraright atrial conduction time from the sinus node to the respective recording area (Figure 1B).

P‐proximal coronary sinus (P‐PCS) interval: Measured from the onset of the P wave to the onset of the earliest reproducible rapid deflection of the atrial electrogram recorded from the proximal poles (most proximal record within the area of the coronary sinus ostium, this position was controlled using as a reference their relationship with HIS catheter and the presence of typical coronary sinus records) of a decapolar catheter (BIOTRONIK) positioned into the coronary sinus to the left lateral portion of the mitral ring (interelectrode distance of the same pair, 5 mm; distance between pairs, 10 mm). This parameter was accepted as a measure of the intraright atrial conduction time from the sinus node to the respective recording area (Figure 1B).

P‐distal coronary sinus (P‐DCS) interval: Measured from the onset of the P wave to the onset of earliest reproducible rapid deflection of the atrial electrogram recorded from the distal poles of a decapolar catheter (BIOTRONIK) positioned into the coronary sinus around the left lateral portion of the mitral ring (interelectrode distance of the same pair, 5 mm; distance between pairs, 10 mm). This parameter was accepted as a measure of interatrial conduction time (Figure 1B).

ΔHIS‐HRA: calculated as the difference of P‐HIS − P‐HRA. It was a way of quantifying the activation difference between two regions within the right atrium.

ΔPCS‐HRA: calculated as the difference of P‐PCS − P‐HRA. It was a way of quantifying the activation difference between two regions within the right atrium.

ΔPCS‐HIS: calculated as the difference of P‐PCS − P‐HIS. It was a way of quantifying the activation difference between two regions within the right atrium.

ΔDCS‐PCS: calculated as the difference of P‐DCS − P‐PCS. It was considered a measure of intraleft atrial conduction time.

Morphology of the electrograms: refers to whether these were biphasic, triphasic, quadriphasic, pentaphasic, or hexaphasic. Morphology was determined for each local electrogram (HRA, HIS, PCS, and DCS).

Electrogram duration: refers to the duration of each local electrogram (HRA, HIS, PCS, and DCS) measured from onset to offset.

The measurements were performed in sinus rhythm by an experienced electrophysiologist, avoiding interobserver error, blinded from the data of each case and with the patient awake, without the effect of anesthetics or isoprenaline, and before the application of radiofrequency, on an EP TRACER (CardioTek) multichannel polygraph. Electrophysiological measurements were made manually using electronic calipers at sweep speed of 300 mm/s, and expressed in milliseconds. The catheter positions were monitored using biplane fluoroscopy with standard right anterior oblique and left anterior oblique views.

The diagnosis of atrioventricular node reentry tachycardia (AVNRT) was confirmed by inducing tachycardia in all cases. The presence of accessory pathways was demonstrated by revealing abnormal retrograde conduction through the pathway through stimulation from the right apex ventricle, and in most cases, orthodromic tachycardia could be induced.

The echocardiographics parameters studied were: interventricular septal thickness in diastole; left atrium size (anteroposterior); left ventricular internal diameter in diastole; left ventricular posterior wall thickness in diastole; right atrium size (minor axis), measured according to current recommendations from the American Society of Echocardiography and the European Association of Cardiovascular Imaging.^5^ The echocardiographic study was performed prior to the electrophysiological study according to the protocol established in the institution.

### 2.2 Statistical processing

For all analyses, commercially available computer software (SPSS Version 21.0, SPSS Inc.) was used. For the comparison of variables with parametric distribution the Student's *t* test was used, for variables with nonparametric distribution the Mann‐Whitney U test was used. The verification of the normal distribution of the data or its absence was determined by the Kolmogorov‐Smirnov test. The comparison of categorical variables was carried out using the chi square test. For the correlations, the Pearson's correlation coefficient was determined, except when the morphology of the electrograms (ordinal qualitative variable) was included, estimating in this case the Spearman correlation coefficient. Multivariate analysis was performed using multiple linear regression. The discriminant capacity of the PWD to find cases with values ≥75 percentile in electrophysiological parameters was determined using ROC (Receiver Operating Characteristic) curves. Values of *P* < .05 were set as the minimum level of statistical significance throughout the study.

### 2.3 Ethical aspects

All patients included in the study gave their informed consent to carry out the electrophysiological study and radiofrequency endocavity ablation. The local ethics committee approved this study.

## 3 RESULTS

General data shows that patients with AVNRT were older than those with accessory pathways. The prevalence of male sex and atrial fibrillation was higher in cases with accessory pathways. High blood pressure was more prevalent in the AVNRT group. No significant differences were found in diabetes mellitus, heart rate, body weight or echocardiographic parameters (Table 1).

**TABLE 1:** Baseline and echocardiographic findings of all patients and stratified by arrhythmic substrates

	All patients n = 153	Arrhythmic substrates		
		AVNRT n = 83	Accessory pathways n = 70	*P*
Demographic				
Age, year	39.53 ± 14.36	43.40 ± 13.83	34.94 ± 13.70	.001^a^
Male sex	58 (37.91)	22 (26.51)	36 (51.43)	.002^a^
AF in the EPS	45 (29.41)	17 (20.48)	28 (40.0)	.026^a^
Comorbidities				
Hypertension	36 (23.53)	27 (32.53)	9 (12.86)	.010^a^
Diabetes mellitus	4 (2.61)	3 (3.61)	1 (1.43)	1.000
Bronchial asthma	8 (5.23)	3 (3.61)	5 (7.14)	.515
Body weight (kg)	69.39 ± 10.01	68.49 ± 9.62	70.50 ± 10.44	.146
Heart rate (bpm)	82.05 ± 20.50	82.55 ± 21.80	81.41 ± 18.86	.737
Echocardiographics findings				
LVEF (%)	59.80 ± 2.81	59.56 ± 2.90	60.11 ± 2.67	.097
LVIDd (mm)	47.14 ± 3.96	47.24 ± 3.92	47.02 ± 4.04	.698
IVSd (mm)	9.56 ± 1.04	9.61 ± 1.04	9.49 ± 1.04	.321
LVPWd (mm)	9.20 ± 1.03	9.32 ± 1.03	9.06 ± 1.01	.089
LA size (mm)	33.65 ± 4.56	33.86 ± 4.36	33.39 ± 4.82	.522
RA size (mm)	27.76 ± 3.81	28.01 ± 3.78	27.45 ± 3.85	.365

Comparison were made between substrates.

Values are presented as mean ± standard deviation or number (%).

Abbreviations: AF, atrial fibrillation; AVNRT, atrioventricular nodal reentrant tachycardia; bpm, beats per minute; EPS, electrophysiological study; IVSd, interventricular septal thickness in diastole; LA, left atrium; LVIDd, left ventricular internal diameter in diastole; LVPWd, left ventricular posterior wall thickness in diastole; RA, right atrium.

^a^ Comparisons with statistical significance.

The Pmax and PWD showed average values that are higher than the normal cutoff points for these variables but with no significant differences between patients with AVNRT and accessory pathways (Table 2).

**TABLE 2:** Characterization of P‐wave parameters in all patients and by arrhythmic substrates

P‐wave parameters	All patients n = 153	Arrhythmic substrates		
		AVNRT n = 83	Accessory pathways n = 70	*P* value
Pmax	122.30 ± 14.56	121.57 ± 13.52	123.11 ± 15.75	.625
Pmin	72.82 ± 13.19	73.47 ± 13.45	72.09 ± 13.00	.829
PWD	49.14 ± 15.51	47.75 ± 14.15	50.70 ± 16.93	.794

Comparison were made between substrates.

Values are presented as mean ± standard deviation.

Abbreviations: AVNRT, atrioventricular nodal reentrant tachycardia; Pmax, maximum P‐wave duration; Pmin, minimum P‐wave duration; PWD, P‐wave dispersion.

**FIGURE 2:**
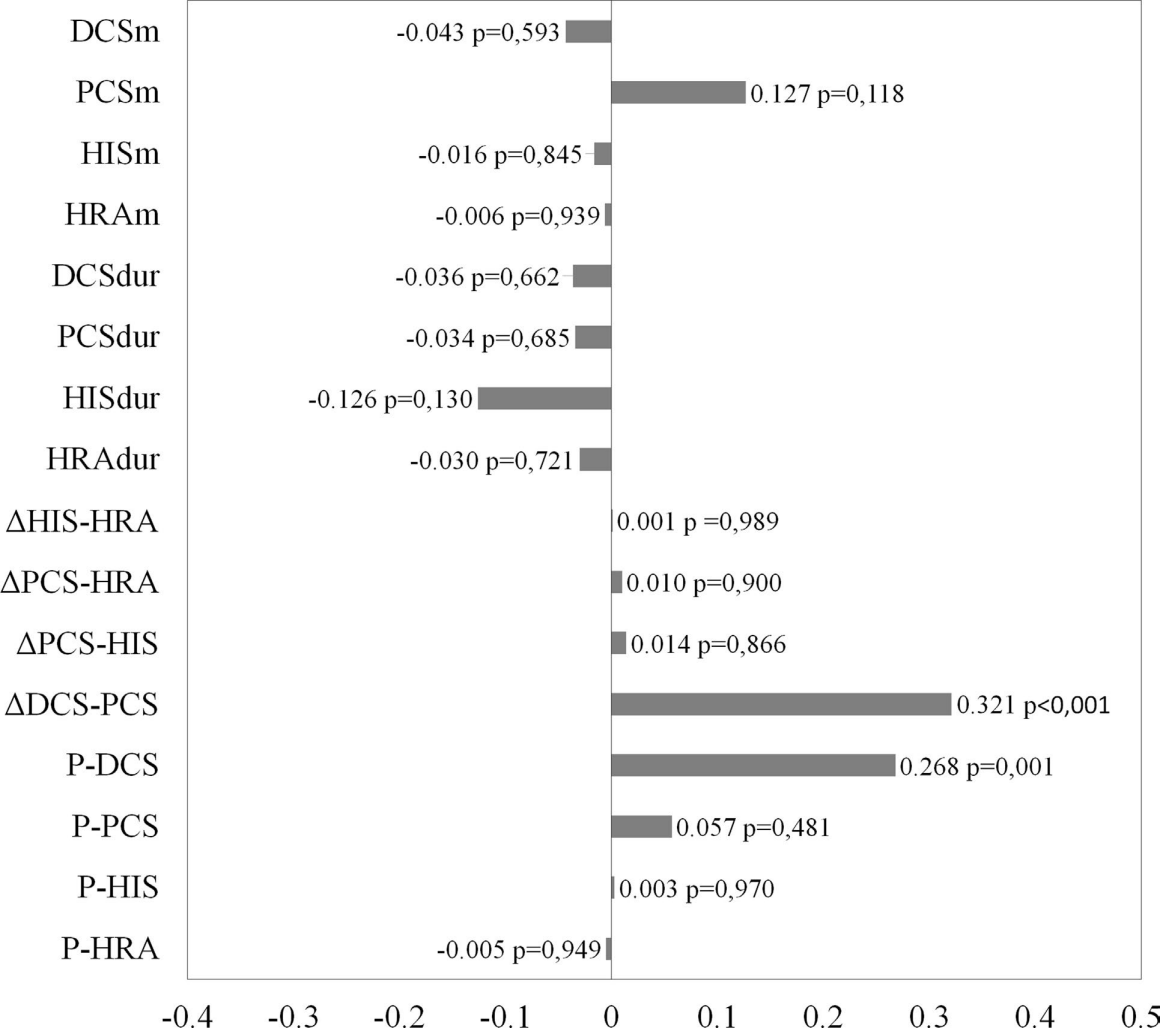
Results of the correlation of all the electrophysiological parameters studied with the P‐wave dispersion. HRAm, HISm, PCSm, DCSm/HRAdur, HISdur, PCSdur, DCSdur: morphology of atrial electrograms/duration of each local electrogram in high right atrium, histogram area, proximal coronary sinus and distal coronary sinus, respectively; P‐HRA, P‐HIS, P‐PCS, P‐DCS (interatrial conduction time): P‐A intervals; ΔHIS‐HRA, ΔPCS‐HRA, ΔPCS‐HIS, ΔDCS‐PCS (intraleft atrial conduction time): activation difference between two regions

Of the 16 variables used to assess atrial conduction only P‐DCS and ΔDCS‐PCS showed a significant correlation with PWD. This correlation was direct and weak (Figure 2). The low coefficients of determination obtained from the simple linear correlation also suggest that P‐DCS (*R* ^2^ = 0.071; 7.1%) and ΔDCS‐PCS (*R* ^2^ = 0.103; 10.3%) weakly explain PWD.

In the multivariable linear regression analysis, ΔDCS‐PCS and P‐DCS were independent predictors of PWD controlling for potential confounders such as age, arterial hypertension, atrial fibrillation during the electrophysiological study, the types of arrhythmic substrates (AVNRT and accessory pathways) and the atrial size (Table 3). Performing the multivariate analysis for arrhythmic substrates, it is observed that only ΔDCS‐PCS continued to be an independent predictor of PWD (Tables 4 and 5).

**TABLE 3:** Multivariable analysis considering separately the atrial conduction times involving the left atrium in all patients

	β coefficient	CI 95%	*P* value
Age	0.130	−0.049‐0.321	.149
ΔDCS‐PCS	0.301	0.278‐0.928	<.001^a^
Hypertension	0.011	−5.741‐6.499	.903
AF	0.073	−2.984‐7.696	.385
AVRNT	0.002	−5.207‐5.349	.979
Accessory pathways	0.011	−1.375‐1.564	.899
LA size	−0.020	−0.594‐0.460	.802
RA size	0.060	−0.398‐0.871	.462

	β coefficient	CI 95%	*P* value
Age	0.139	−0.044‐0.334	.131
P‐DCS	0.247	0.083‐0.526	.007^a^
Hypertension	−0.008	−6.508‐5.928	.927
AF	0.032	−4.900‐6.989	.729
AVRNT	−0.017	−5.904‐4.907	.856
Accessory pathways	−0.015	−1.626‐1.364	.863
LA size	−0.023	−0.613‐0.461	.781
RA size	0.048	−0.456‐0.836	.561

Abbreviations: AF, atrial fibrillation in the electrophysiological study; AVNRT, atrioventricular nodal reentrant tachycardia; CI, confidence interval; LA, left atrium; P‐DCS, interatrial conduction time; RA, right atrium; ΔDCS‐PCS, intraleft atrial conduction time.

^a^ Only significant variable in the analysis.

^b^ The regression analysis did not include P‐DCS and ΔDCS‐PCS together because it weakened and lost statistical significance.

**TABLE 4:** Multivariable analysis considering separately the atrial conduction times involving the left atrium in patients with atrioventricular nodal reentrant tachycardia

	β coefficient	CI 95%	*P* value
Age	0.105	−0.138‐0.350	.389
ΔDCS‐PCS	0.233	0.028‐1.005	.039^a^
Hypertension	0.011	−6.844‐7.486	.929
AF	0.002	−7.331‐7.498	.982
LA size	0.051	−0.556‐0.890	.647
RA size	−0.072	−1.103‐0.558	.515

	β coefficient	CI 95%	*P* value
Age	0.136	−0.104‐0.381	.259
P‐DCS	0.215	−0.017‐0.518	.066
Hypertension	−0.007	−7.377‐6.972	.955
AF	−0.052	−9.545‐6.030	.654
LA size	0.047	−0.573‐0.882	.674
RA size	−0.063	−1.075‐0.600	.574

Abbreviations: AF, atrial fibrillation in the electrophysiological study; CI, confidence interval; LA, left atrium; P‐DCS, interatrial conduction time; RA, right atrium; ΔDCS‐PCS, intraleft atrial conduction time.

^a^ Only significant variable in the analysis.

^b^ The regression analysis did not include P‐DCS and ΔDCS‐PCS together because it weakened and lost statistical significance.

**TABLE 5:** Multivariable analysis considering separately the atrial conduction times involving the left atrium in patients with accessory pathways

	β coefficient	CI 95%	*P* value
Age	0.143	−0.129‐0.466	.262
ΔDCS‐PCS	0.357	0.189‐1.128	.007^a^
Hypertension	0.017	−11.399‐12.988	.897
AF	0.128	−4.174‐12.452	.323
LA size	−0.118	−1.183‐0.398	.325
RA size	0.226	−0.067‐1.954	.067

	β coefficient	CI 95%	*P* value
Age	0.121	−0.173‐0.457	.371
P‐DCS	0.264	−0.068‐0.761	.100
Hypertension	0.010	−12.163‐13.156	.938
AF	0.108	−6.712‐13.718	.496
LA size	−0.109	−1.184‐0.459	.381
RA size	0.191	−0.242‐1.839	.130

Abbreviations: AF, atrial fibrillation in the electrophysiological study; CI, confidence interval; LA, left atrium; P‐DCS, interatrial conduction time; RA, right atrium; ΔDCS‐PCS, intraleft atrial conduction time.

^a^ Only significant variable of the model.

^b^ The regression analysis did not include P‐DCS and ΔDCS‐PCS together because it weakened and lost statistical significance.

**FIGURE 3:**
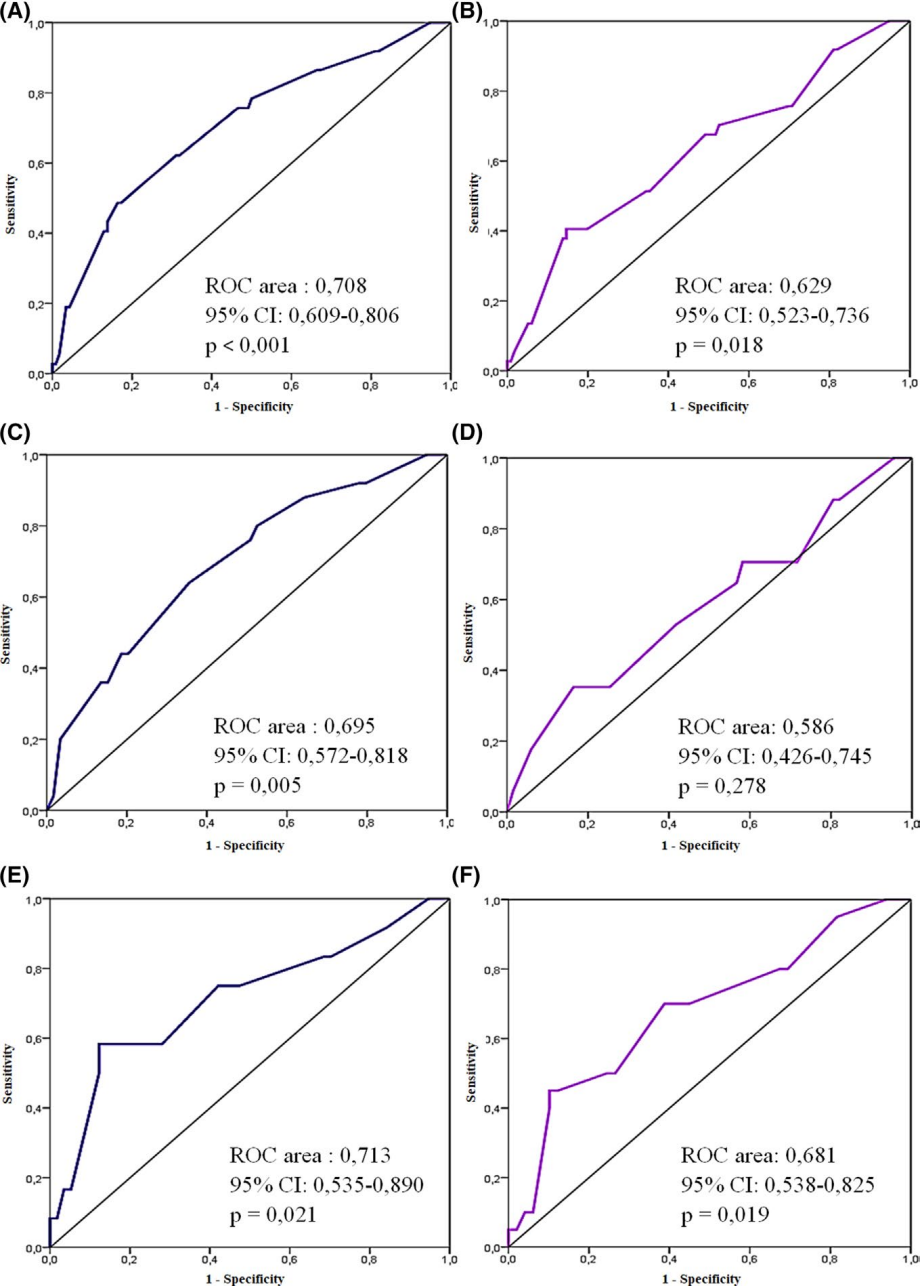
ROC (receiver operating characteristic) curves showing the relationship between sensitivity and specificity through all the possible P‐wave dispersion values that define patients with ΔDCS‐PCS (blue curves) and P‐DCS (mauve curves) ≥75 percentile. A and B: in all patients; C and D: in patients with AVNRT; F and G: in patients with accessory pathways. AVNRT, atrioventricular nodal reentrant tachycardia; ΔDCS‐PCS, intraleft atrial conduction time; P‐DCS, interatrial conduction time

The ROC analysis showed that regardless of the types of arrhythmic substrates, PWD discriminates patients with P‐DCS (PWD cutoff value: 47 ms, sensitivity: 67.6%, specificity: 50.9%) and ΔDCS‐PCS (PWD cutoff value: 45.5 ms, sensitivity: 75.7%, specificity: 52.6%) ≥75 percentile (Figure 3A,B).

When the analysis by arrhythmic substrates was performed, it was found that in the cases with AVNRT, the PWD had discriminant power to find cases with ΔDCS‐PCS ≥75 percentile (PWD cutoff value: 51.0 ms, sensitivity: 64.0%, specificity: 64.4%), but not with P‐DCS intervals ≥75 percentile (Figure 3C,D). In the case of patients with accessory pathways, PWD retained its discriminating ability to find cases with a P‐DCS interval (PWD cutoff value: 47.0 ms, sensitivity: 70.0%, specificity: 61.2%) and ΔDCS‐PCS (PWD cutoff value: 47.0 ms, sensitivity: 75.5%, specificity: 58.8%) ≥75 percentile (Figure 3E,F).

## 4 DISCUSSION

The mean values of PWD and Pmax are increased in our series with respect to the reported upper cutoff points for these parameters. Analyzing 295 patients randomly selected from the original cohort of the Framingham study with a wide age range and without cardiovascular disease or hypertension, diabetes mellitus or obesity, Magnani et al^6^ determined that the median PWD was 34 ms with a range between the 25‐75 percentile of 28‐43 ms. Pérez‐Riera et al^3^ consider that the normal value is between 20 and 38 ms with a mean of 29 ms, almost equal to that reported in the control group used by Dilaveris et al^1^ that presented a range of 21‐35 ms (mean: 28 ms). The P wave is prolonged when its duration is >120 ms.^7^ A study comparing PWD and Pmax in patients with AVNRT with (35.1/108.8 ms) and without (27.9/100.2 ms) previous history of paroxysmal atrial fibrillation found no elevated mean values for these parameters.^8^ The occurrence of frequent episodes of tachycardias could be an inducer of electrical remodeling in the studied patients, which could partly explain our observations.

PWD is considered by many to be an electrocardiographic parameter originating from regional differences in atrial conduction, but the evidence supporting such an assertion derives primarily from echocardiographic techniques (tissue doppler, strain rate) that are approximations of true electrophysiological measurements, although such techniques have been validated.^9^, ^10^, ^11^

Demir et al^12^ found that in patients with type 2 diabetes mellitus PWD was directly and significantly correlated (*r* = .429; *P* < .001) with the interatrial electromechanical delay estimated by tissue Doppler. In hypertensive patients with no history of atrial fibrillation, PWD was found to be an independent predictor of intraleft atrial conduction time (B = 0.306; *P* = .044) and interatrial conduction time (B = 0.483; *P* = .05) measured by tissue Doppler, in addition to showing direct and significant correlations with interatrial (*r* = .722), intraright (*r* = .643) and intraleft (*r* = .722) atrial conduction times.^13^ Ermis et al^14^ also found that PWD was significantly correlated with intra‐atrial and interatrial conduction times in prehypertensive patients, also using the same measurement method. In a case‐control study conducted in healthy nonsmokers and smokers, significant correlations were obtained between PWD and interatrial electromechanical delay (*r* = .653; *P* = .001),^15^ a finding that has also been documented in hypertensive patients (*r* = .72; *P* < .001).^16^ Even in pregnant women with preeclampsia, PWD and interatrial (*r* = .46; *P* < .001) and intra‐atrial (*r* = .39; *P* < .001) electromechanical delay have been shown to correlate directly and significantly.^17^ There is a significant correlation between PWD and the interatrial electromechanical delay interval (*r* = .54; *P* < .01) in patients with polycystic ovary syndrome.^18^

Badran et al^19^ using 2D‐strain rate in patients with idiopathic dilated cardiomyopathy found that PWD is directly correlated in these patients (*r* = .45, *P* < .00001) with the quantification of the electromechanical delay of the left atrium, but this finding was not found in the control group. In addition, higher values of PWD and of the interatrial, right atrial and left atrial electromechanical delay times measured by 2D strain rate have been found in nondipper hypertensive patients compared to dipper.^20^

The previous studies show that there is a relationship between PWD and atrial conduction, which coincides with our results that also demonstrate this relationship, which occurs mainly with electrophysiological parameters that involve the left atrium. Although we analyzed 16 variables that evaluate atrial conduction, only P‐DCS and ΔDCS‐PCS are related to PWD, and predict it. These data suggest that PWD may be preferentially explained by changes in left atrial conduction and/or interatrial conduction. PWD was better related to ΔDCS‐PCS than P‐DCS, a parameter that constitutes a measure of left intraatrial conduction time. No conduction parameters confined to the right atrium had any effect on PWD.

Previous research has found moderate to strong relationships between atrial conduction and PWD.^12^, ^13^, ^15^, ^16^, ^17^, ^18^, ^19^ However, in our study they were weak as observed in the correlation coefficients obtained. Furthermore, in the multivariate analysis we also obtained lower beta coefficients than in the study by Djikic et al,^13^ although a limitation in this comparison could be the different ways of measuring atrial conduction times and the different study populations. The discriminatory capacity of the PWD to find patients with high values of P‐DCS and ΔDCS‐PCS, although significant, was modest considering the observed sensitivity and specificity values, which supports the aforementioned. Putting all this evidence together, we consider that atrial conduction only weakly explains PWD.

## 5 CONCLUSIONS

Interatrial and intraleft atrial conduction times were directly and significantly correlated with PWD, but weakly, and were independent predictors of PWD. In general, PWD correctly discriminates patients with high values in interatrial and intraleft atrial conduction times, but moderately. This is maintained in cases with accessory pathways, but in patients with AVNRT it only does so for intraleft atrial conduction times. Interatrial and intraleft atrial conduction times weakly explains PWD.

## CONFLICT OF INTEREST

The authors declare no conflict of interest for this article.
